# Retrospection-Simulation-Revision: Approach to the Analysis of the Composition and Characteristics of Medical Waste at a Disaster Relief Site

**DOI:** 10.1371/journal.pone.0159261

**Published:** 2016-07-14

**Authors:** Li Zhang, Lihua Wu, Feng Tian, Zheng Wang

**Affiliations:** 1 Institute of Medical Equipment, Academy of Military Medical Sciences, Tianjin, China; 2 Basic Courses Department, Logistics University of the Chinese People’s Armed Police Forces, Tianjin, China; Charles University in Prague, CZECH REPUBLIC

## Abstract

A large amount of medical waste is produced during disaster relief, posing a potential hazard to the habitat and the environment. A comprehensive understanding of the composition and characteristics of medical waste that requires management is one of the most basic steps in the development of a plan for medical waste management. Unfortunately, limited reliable information is available in the open literature on the characteristics of the medical waste that is generated at disaster relief sites. This paper discusses the analysis of the composition and characteristics of medical waste at a disaster relief site using the retrospection-simulation-revision method. For this study, we obtained 35 medical relief records of the Wenchuan Earthquake, Sichuan, May 2008 from a field cabin hospital. We first present a retrospective analysis of the relief medical records, and then, we simulate the medical waste generated in the affected areas. We ultimately determine the composition and characteristics of medical waste in the affected areas using untreated medical waste to revise the composition of the simulated medical waste. The results from 35 cases showed that the medical waste generated from disaster relief consists of the following: plastic (43.2%), biomass (26.3%), synthetic fiber (15.3%), rubber (6.6%), liquid (6.6%), inorganic salts (0.3%) and metals (1.7%). The bulk density of medical relief waste is 249 kg/m^3^, and the moisture content is 44.75%. The data should be provided to assist the collection, segregation, storage, transportation, disposal and contamination control of medical waste in affected areas. In this paper, we wish to introduce this research method of restoring the medical waste generated in disaster relief to readers and researchers. In addition, we hope more disaster relief agencies will become aware of the significance of medical case recording and storing. This may be very important for the environmental evaluation of medical waste in disaster areas, as well as for medical waste management and disposal.

## 1. Introduction

**Fig 1:**
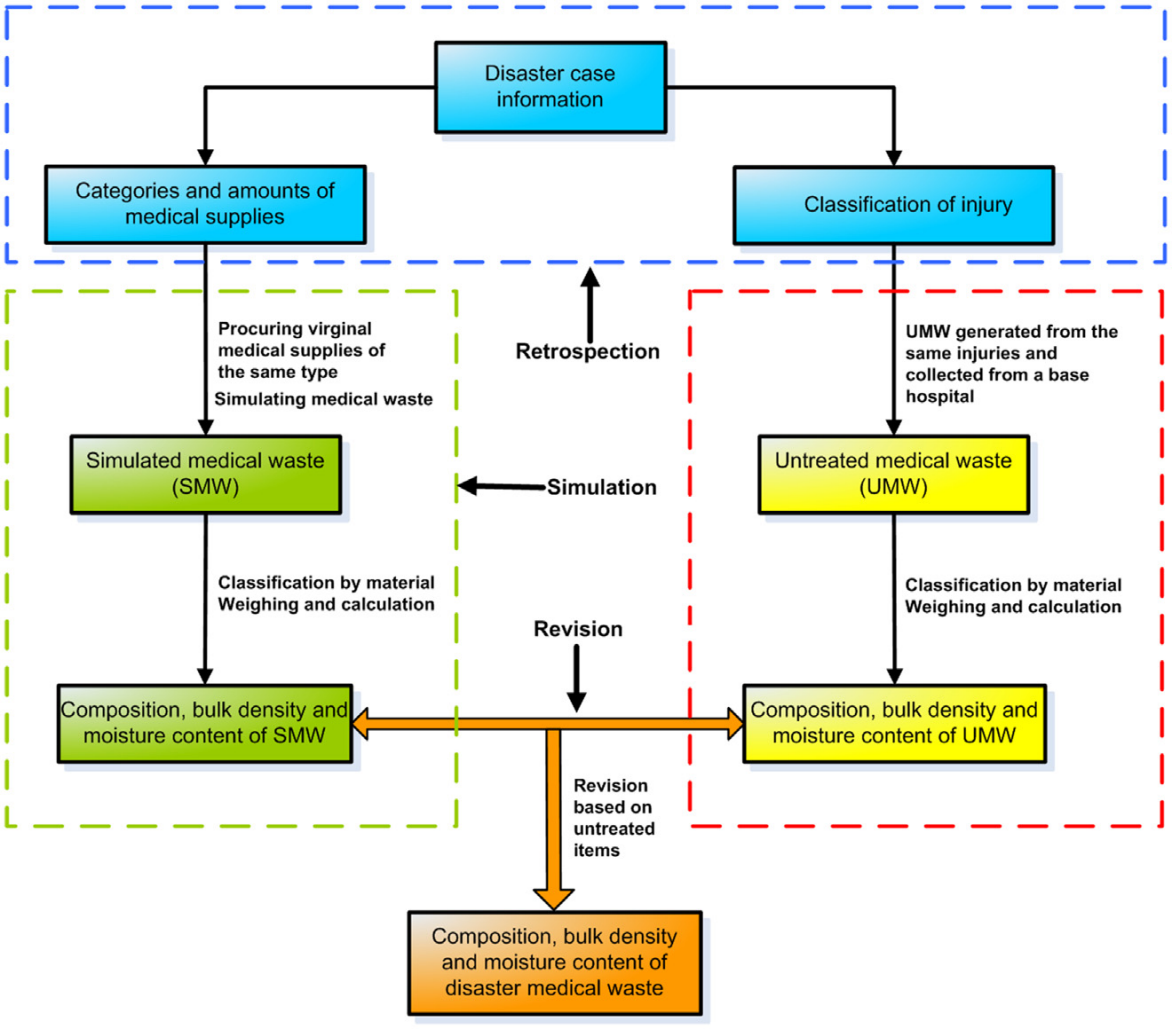
Schema of RSR.

Geological disasters have frequently occurred all over the world in recent years. Handling medical waste generated from medical aid units under disaster emergency relief conditions has become one of the most heavily researched areas of international science, technology and environmental protection [1]. Inadequate disposal of medical waste in stricken areas may cause infection and pose serious threats to human health and environmental safety or may even cause infection or increase the prevalence of infectious diseases and introduce new difficulties for relief efforts [2, 3]. To avoid the dissemination and diffusion of pose-disaster epidemics and to prevent secondary environmental disasters [4], safe handling of medical waste in stricken areas is required [1, 5]. A comprehensive understanding of the composition and characteristics of medical waste is one of the most basic steps in the development of a plan for medical waste disposal. However, the analysis of the composition and characteristics of medical waste in earthquake-stricken areas has not been reported in the literature to date. Due to the influence of different sources, medical waste varies in variety and quantity [6–15]. This paper discusses the analysis of the composition and properties of medical waste at a disaster relief site using the retrospection-simulation-revision (RSR) method. The RSR approach proceeds as follows: (i) conduct a retrospective analysis of disaster relief information. This step includes calculating the medical supplies actually used by the medical relief unit according to the raw medical records. (ii) According to the medical supply data, create the simulated medical waste (SMW), which is similar to that at the disaster relief site. (iii) Analyze the injury types of the medical relief cases in stricken areas and collect the untreated medical waste (UMW) produced by similar cases generated from base hospitals. (iv) Determine the composition and characteristics of medical waste in stricken areas using the composition and characteristics of UMW to revise those of SMW. Fig 1 is the schema of RSR.

A devastating earthquake of magnitude 8.3 Ms occurred on May 12, 2008 in Wenchuan County, China, resulting in widespread infrastructure destruction as well as human casualties. During the emergency response period, the production of medical waste increased dramatically to approximately 126 t/d, and 70% of this waste was from front-line medical relief units and field cabin hospitals in remote mountain areas [16]. When a disaster occurs, due to the shortage of disaster relief personnel and materials and due to the initial focus on saving lives, it is usually unfeasible to organize personnel to sample and analyze UMW in disaster areas in a timely manner. Medical institutions throughout China organized relief teams after the Wenchuan Earthquake to participate in the medical treatment of wounded persons and disease prevention. The RSR approach was applied to the medical waste generated from a medical relief unit in response to the 2008 Wenchuan Earthquake. This work will provide predictable data on waste composition and characteristics for assisting the scientific disposal of medical waste in stricken areas.

## 2. Materials and Methods

### 2.1. Data collection

For comparison, a search for published data concerning medical waste streams and components was attempted. During the collection process of rescue cases, many disaster relief agencies were contacted. Unfortunately, most disaster relief agencies have few effective medical records when implementing medical rescues in a disaster. However, 35 copies of medical records were collected for this study. Data was also collected from hospital reports of waste streams and disposal and from the open literature [6–12, 17–19] on the characteristics of the various types of wastes that are generated in health facilities. The generation of medical waste produced from hospitals or medical facilities was analyzed using the various data collection methods previously mentioned. However, unfortunately, it is very difficult to directly compare the data in this study to that obtained from other studies on medical waste because the classification methods varied among hospitals or among studies.

This research is based on the 35 medical relief records of the Wenchuan Earthquake in Sichuan in May 2008 form a field cabin hospital belonging to the 255th hospital of the Chinese People^’^s Liberation Army (PLA). The 255th hospital is a base hospital located in Tangshan, Hebei Province. This field cabin hospital is mobile and can provide medical assistance during a disaster. During the collection of the 35 medical records, the information management department of the 255th hospital encrypted the patient's name, birth date, identification (ID) card number and medical record number, and the medical records provided for this study only included the patient's gender, age, diagnostic result and treatment plan. The ethics committee of the Academy of Military Medical Sciences previously approved this research (S1 and S2 Figs). The 35 medical records were collected as a case study to characterize the medical waste at a disaster relief site.

### 2.2. Sample preparation

According to the amounts of medical supplies actually used in the 35 medical records, the SMW, which is similar to that at the disaster relief site, was performed. All SMW consisted of unused, disposable medical supplies.

In accordance with the season of the earthquake, this study considered well-directed UMW generated from similar cases in the operating rooms and emergency rooms of the 255th hospital during May and June of 2015. Grab samples were collected for each similar case, and after each treatment, the samples were well mixed to obtain a homogenous mixture. The samples were stored at approximately 4°C in cooler bags filled with ice cubes to prevent degradation.

**Fig 2:**
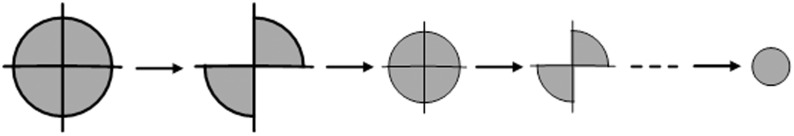
Schema of quartering.

The final experimental samples of UMW and SMW were prepared by quartering [20]. The medical waste was stirred to collect them into a circle, and then, the circle was quartered. Then, two parts at the two opposite angles were randomly discarded, and the remaining parts were flattened and quartered again (Fig 2). This time, half of the sample was discarded, and after splitting 3 times, the SMW sample weighed 12.63 kg, and the UMW sample weighed 19.57 kg.

### 2.3. Bulk density analysis

The bulk density determination of SMW and UMW was performed according to the standard method [20] in which (i) the trash can that contains medical wastes with an effective volume of 0.050 m^3^ and that is constructed of high density polyethylene is weighed; (ii) a sample is placed in the trash can, and it is vibrated three times for five seconds each time with no compaction; (iii) more sample is added and vibrated three times for five seconds each time with no compaction; this process is repeated until the trash can is filled with the medical waste; (iv) the sample (including the weight of the trash can) is weighed; and (v) the weight is determined five times by repeating steps (ii)-(iv). The bulk density is then calculated according to formula (1):

$$\text{d}=\frac{1000}{\text{m}}\sum_{\text{j}=1}^{\text{m}}\frac{{\text{M}}_{\text{j}}-\text{M}}{\text{V}}$$ (1)

where *d* is the bulk density of the sample in kilograms per cubic meters (kg/m^3^), *m* is the number of determinations, *j* specifies the ordinals of weighing, *M* is the weight of the empty trash can in kilograms (kg), *M*_*j*_ is each weight (including the weight of the trash can) in kilograms (kg) and *V* is the trash can volume in cubic meters (m^3^) (the bulk density data are in the S1 Table).

### 2.4. Composition analysis

After determing the bulk density of the SMW and UMW samples, objects with large particle sizes in the SMW and UMW samples are ground to 100 mm and smaller, and then, they are spread onto a clean, flat, non-absorbent board. The analysis of the compositions of SMW and UMW was performed according to the standard method [20] in which (i) the SMW and UMW samples were weighed; (ii) the SMW and UMW samples were sorted by category; (iii) the categories of the same material component were consolidated, and each component was weighed. For items composed of a variety of materials, those containing easily determined components that can be disassembled are classified into the appropriate categories by their materials; those for which it is difficult to determine the components and disassemble into the appropriate categories by their principle materials are also classified; and (iv) the percentage of components is calculated using formula (2):

$$C_{\text{i}}=\frac{M_{\text{i}}}{M}\times 100$$ (2)

where *C*_i_ is the wet basis content of a component (%), *M*_i_ is the wet weight of a component in kilograms (kg) and *M* is the sample wet weight in kilograms (kg) (the composition data are in S2 Table).

### 2.5. Moisture content analysis

The moisture content determination of SMW and UMW was performed according to the standard method [20] in which (i) different component test samples of medical waste were placed in a dry container, and their weight was taken and recorded; (ii) the samples were placed in a heated blower thermostat dryer for 8 h at 105°C, and then, they were weighed after cooling for 0.5 h; (iii) the drying step was repeated for 1–2 h, and the samples were weighed after cooling for 0.5 h. The samples were not considered completely dry until the difference between the two weights was less than 1% of the sample; and (iv) the moisture content of the samples was calculated using formulas (3) and (4):

$$C_{i\left(w\right)}=\frac{M_{i}-M_{i}^{,}}{M_{i}}\times 100$$ (3)

$$C_{\left(\text{w}\right)}=\sum_{i=1}^{\text{n}}C_{i\left(w\right)}\times \frac{C_{\text{i}}}{100}$$ (4)

where *C*_*i*(*w*)_ is the moisture content of a component (%), *C*_(w)_ is the comprehensive moisture content (%), $M_{i}^{,}$ is the dry weight of a component in grams (g), *i* specifies the ordinals of all components and n is the number of compositions.

## 3. Results and Discussion

### 3.1. Biological characteristics of medical waste

The potential microbiological risks associated with medical waste are still unfamiliar to healthcare workers because the literature on the role of infectious medical waste as a reservoir of diseases is extremely limited. There are few reports documenting the infectious risks of medical waste management, and unfortunately, scientifically substantiated evidence on the actual content of microorganisms, the survival of microorganisms in medical waste and the infectious risks to healthcare workers and the general public is extremely rare [19].

Medical waste consists of two types: infectious waste and non-infectious waste. Mohee [21] found that approximately 90% of medical waste was non-infectious and was similar in properties to domestic waste. The remaining 10% was infectious hazardous waste. Townend [22] found that 10–25% of healthcare waste was termed as infectious, pharmaceutical, radioactive and chemical waste, which may pose a variety of health and environmental risks. In France, 15–20% of medical waste is infectious waste [21], while in the USA, approximately 15% is considered infectious waste [23].

The infectious risk posed by medical waste to human health and the environment is the potential presence of pathogenic microorganisms; this risk still requires evaluation. Medical waste may contain a great variety of pathogenic microorganisms. Individuals involved in the treatment of clinical waste are exposed to infectious agents through several routes, including skin penetration and skin contact, or via the aerogenic route [22]. Park et al. [24] investigated the types of microbial agents in various medical waste. Many (opportunistic) pathogenic bacteria, including *Pseudomonas* spp., *Lactobacillus* spp., *Staphylococcus* spp., *Micrococcus* spp., *Kocuria* spp., *Brevibacillus* spp., *Microbacterium oxydans*, and *Propionibacterium acnes*, were identified from the various medical waste. Commonly identified bacterial and viral pathogens, such as *Pseudomonas* spp., *Corynebacterium diphtheriae*, *Escherichia coli*, and *Staphylococcus* spp., as well as respiratory synctial virus (RSV), were inoculated into either gauzes or diapers. The health risk associated with medical waste could be minimal if medical waste is properly managed. However, the effective management and safe disposal of these wastes are still necessary.

### 3.2. Retrospective analysis of medical relief cases

A retrospective analysis of the medical relief records was performed by physicians who participated in the medical relief effort in this earthquake. The data were analyzed anonymously. After performing the analysis, physicians presented an injury classification of the 35 medical relief records, as shown in Table 1. The numbers of the medical supplies actually used according to the medical records are reported in Table 2. Thirty-six categories of medical supplies were used in treatment (Table 2). According to the standard method [20], all medical supplies fall into six major categories according to material (Table 3).

**Table 1:** Injury classification of 35 medical relief records.

Injury type	Head and neck injuries		Limb fractures		Thoraco-abdominal injuries		Fatigue shock	Total
	Operation	Emergency debridement	Operation	Emergency reduction and fixation	Operation	Emergency debridement		
Number of cases	5	2	5	3	14	0	6	35

**Table 2:** Number of medical supplies used in 35 medical relief records.

Product name	Number of supplies	Product name	Number of supplies	Product name	Number of supplies
Sterile towels	35	Disposable syringes	112	Knife blades	27
Disposable bed sheets	35	Disposable infusion sets	65	Indwelling needles	25
Surgical drapes	24	All types of liquid medicines in plastic bags	182	Arterial blood -taking needles	28
Disposable surgical gowns	97	All types of liquid medicines in plastic bottles	142	Electrodes	120
Disposable surgical caps	97	Drainage bags	11	Plaster bandages	8
Disposable masks	119	Negative pressure drainage apparatus	1	Liquid medicines in ampules	128
Sutures	79	Disposable endotracheal intubations	7	Disposable dressing packs	6
Cotton gauze pads	73	Double -lumen central venous catheters	7	Disposable urinary catheter packs	20
Absorbent cotton balls	112	Tees	17	Disposable wrapping cloth	52
Cotton swabs	190	Gags	7	Lumbar anesthesia puncture sets	16
Abdominal belts	8	Airways	7	Disposable intestinal coinciding equipment	2
Adhesive plasters	25	Rubber surgical gloves	119	All types of wound dressings	52

**Table 3:** Medical waste classification by material.

No.	Material classification	Major medical supplies
1	Synthetic fibers	Disposable bed sheets, treatment towels, surgical gowns, surgical caps, masks, surgical drapes, sutures
2	Biomass	All types of cotton gauze dressings, absorbent cotton balls, cotton swabs, paper supplies, abdominal belts
3	Plastic	Disposable syringes, infusion sets, all types of liquid medicine in plastic bags (bottles), drainage bags, negative pressure drainage apparatus, all types of plastic cannulas, catheters and gags, all types of plastic bags, disposable plastic trays
4	Rubber	Rubber surgical gloves, rubber plugs of all types of medicine liquid bottles
5	Inorganic salts	Plaster bandages, all types of liquid medicine in ampoules
6	Metals	Surgical knife blades, needles, aluminum seals of medicine liquid bottle

The data in Table 1 show that the medical treatment at earthquake disaster sites focuses on emergency site operation and emergency debridement. The relief environment of major natural disasters is completely different from that of a normal hospital, and the various medical supplies taken by medical relief units into stricken areas are light weight, small, portable and non-friable. These supplies of choice are obviously different from materials used in the base hospital. For example, liquid medicines used in base hospitals are glass-packaged products, whereas liquid medicines in plastic packaging (see Table 2) are chosen for relief efforts because glass-packaged products are friable, and their bulk density is considerably greater than that of plastic. For the same amount of liquid medicines, the weight of glass-packaged products is considerably higher than that of products packaged in plastic. Therefore, our research could not be based on the regular medical wastes from the base hospitals. The study on the composition of medical waste in stricken areas should focus on the analysis of medical relief records in stricken areas, supplemented by the analysis of untreated waste in base hospitals.

### 3.3. Comparison between the compositions of SMW and UMW

**Fig 3:**
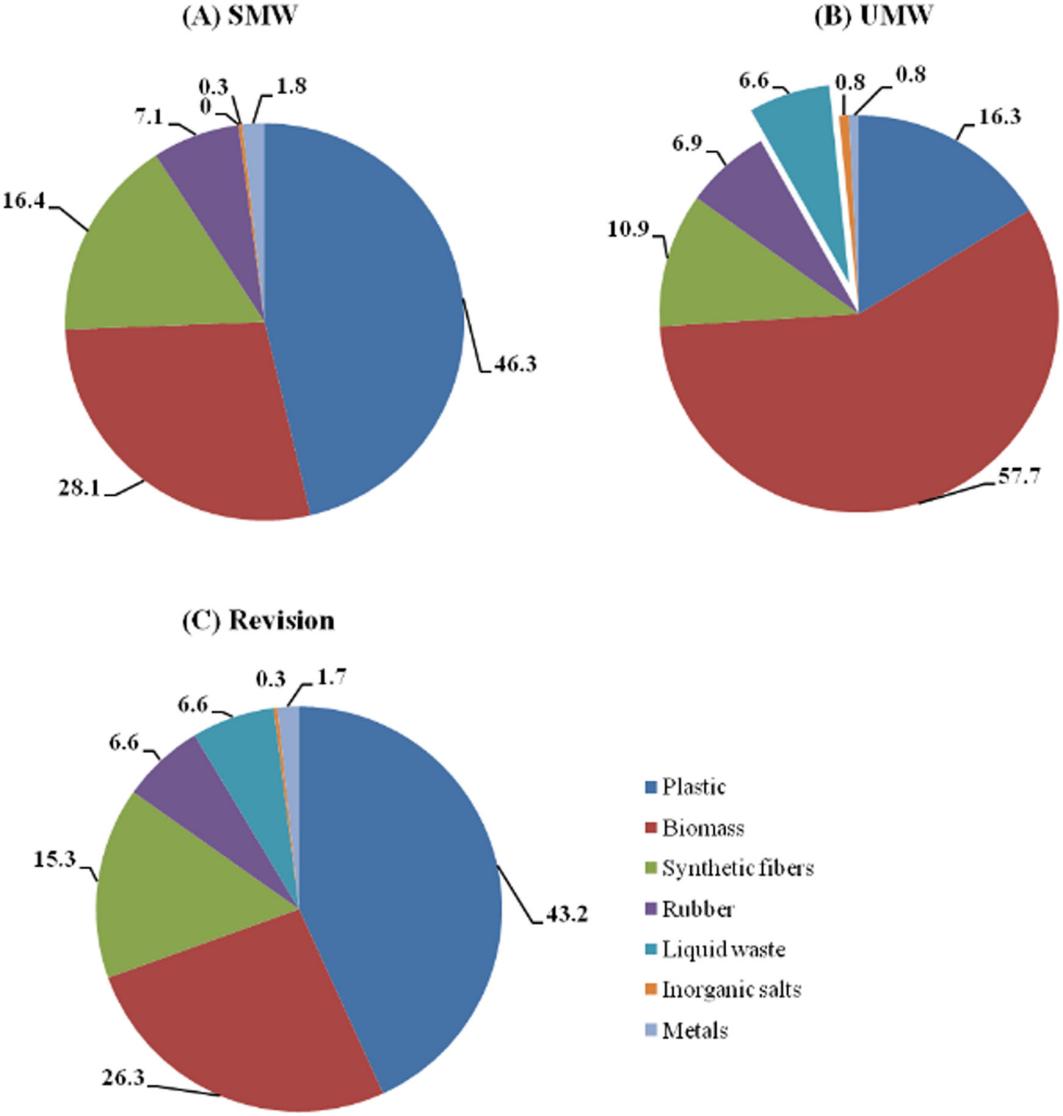
Composition of medical waste (% mass on a wet basis). (A) The composition of SMW (% mass on a wet basis). (B) The composition of UMW (% mass on a wet basis). (C) The composition of Revision that was determined by introducing 6.6% liquid waste, which equals the liquid waste content of UMW, into the SMW and revising the proportions of the other SMW components (% mass on a wet basis).

The compositions of SMW, UMW and the revised simulated medical waste are shown in Fig 3. Plastic waste (46.3%) dominated the SMW composition. Biomass (28.1%) and synthetic fibers (16.4%) were also important SMW fractions. The results in the composition of UMW show values of 16.3% plastic waste, 57.7% biomass and 10.9% synthetic fibers. In addition, the main difference between the SMW and UMW is related to the liquid waste. Because the SMW is composed of unused disposable medical supplies, it does not contain liquid waste components. However, the UMW analysis results show that the amount of medical liquid waste reaches 6.6%.

In the process of being used, medical supplies may be contaminated by various liquid waste, such as human blood, medical solutions and the flushing fluid used to clean wounds. The SMW sample is composed of unused medical supplies. Therefore, it cannot be used to evaluate the content of the liquid waste. The content of liquid waste will directly affect the study on the scientific disposal process of medical waste [25, 26]; therefore, it is an integral part of medical waste composition. To solve this problem, we collected the UMW produced in similar cases from the base hospitals. According to the percentage of liquid waste, we revised the results of SMW using formula (5). In the formula, the liquid waste in UMW accounted for 6.6%. Although this value was not the true proportion of the liquid waste produced by the 35 cases generated from the disaster relief, it was much more approximate than the true value. Thus, we directly introduced this data into the composition of the SMW and revised the proportions of the other SMW components. Compared with the compositions of SMW and UMW, the revision data are much more approximate to the true composition (revision in Fig 3) of medical waste generated according to the 35 records at the disaster relief site.

$$C_{mi}=\frac{C_{i}\times \left(100-6.6\right)}{100}$$ (5)

Here, *C*_*m*i_ is the revised content of a component (%) and *i* specifies the ordinals of the components.

In Fig 3, the composition of the revised simulated medical waste shows that the medical relief waste of the 35 medical cases primarily consisted of plastic (43.2%), biomass (26.3%) and synthetic fibers (15.3%). Among these components, the amount of plastic is greater than that of biomass and synthetic fibers, primarily because most medical supplies taken by medical relief units into stricken areas are products packaged in plastic, and the UMW generated from base hospitals as well as most liquid medicines are glass-packaged products. The data in Fig 3 show that the proportion of biomass (57.7%) in UMW is higher than the proportion (26.3%) in revised simulated medical waste. Biomass components primarily include cotton gauze pads, cotton balls, cotton swabs and abdominal belts, which have good moisture absorption. The percentage of the wet basis of most waste cotton gauzes and dressings of UMW naturally increases after they absorb liquid waste. In the revised data, the sources of data are SMW, and the cotton dressings are unused; thus, the percentage on a wet basis is much lower than that of UMW. There is no significant difference in the percentages of rubber, inorganic salts and metals between the compositions of UMW and the revised simulated medical waste.

### 3.4. Comparison between the bulk densities of SMW and UMW

The bulk density of medical waste is the mass of waste occupying a known volume of medical waste in the natural state. Bulk density is generally reported as mass per unit of volume, e.g., kg/m^3^. In medical waste management, it is important to know the bulk density of the medical waste or the components of the medical waste for many purposes, including the determination of storage space, the size definition for the collection vehicle and the estimation of the requirements for processing equipment (compaction, size reduction, disinfection and others).

The results of the analyses to determine the bulk densities of SMW and UMW reveal that the bulk density for SMW was 181 kg/m^3^, while that for UMW was 249 kg/m^3^. The factors affecting the bulk density of medical waste in stricken areas primarily include the compositions and the degree of compaction of medical waste. The primary reason for the difference between the bulk densities of SMW and UMW is their differential compositions under the same degree of compaction. The data in Fig 3 show that the sum of the inorganic components (liquid waste, inorganic salts and metals) of SMW is 2.1%, and its bulk density is 181 kg/m^3^; while the sum of the inorganic components of UMW is 8.2%, and its density is 249 kg/m^3^. Medical waste bulk density is positively correlated with the content of inorganic components, and the inorganic component increases with bulk density.

The value of the bulk density of SMW is less than the actual value because it does not contain liquid waste. Because the composition of SMW is completely different from that of UMW, we could not find a suitable method for data revision. However, the UMW samples are from similar disease cases; thus, it is assumed that the actual bulk density of the medical waste generated from the relief unit should be close to that of UMW, which is 249 kg/m^3^.

If possible, the institutions that manage medical waste should attempt to obtain additional information on the characteristics of the medical waste that require treatment, primarily to determine the most appropriate method for the materials. The medical waste characteristics are important to define the specific type of equipment required for the treatment.

### 3.5. Comparison between the moisture contents of SMW and UMW

The moisture content of medical waste is one of the key parameters in the waste disposal process and directly affects the calorific value of waste [27]. The presence of moisture will reduce the low calorific value of medical waste and make waste disposal more difficult. Moreover, too much moisture will affect waste transportation, and the increased moisture of medical waste will cause rapid reproduction and spread of bacteria and sources of infection, thus necessitating the determination of the moisture content of medical waste in stricken areas.

The moisture contents of SMW and UMW are given in Table 4. The data in Table 4 show that the moisture content for SMW was 1.50%, while that for UMW was 44.75%. Thus, there is a significant difference in the moisture contents of SMW and UMW. The liquid waste contents are often the key reason for the difference between the moisture contents of SMW and that of UMW. All types of disposable medical supplies in use will carry patient body fluids and liquid medicines, and their residue will increase the moisture content of UMW. However, SMW is composed of unused disposable medical supplies; thus, it does not contain medical residues generated in use. In addition, the data in Table 4 show that the moisture contents of the components in UMW are much higher than those of SMW. The moisture contents in the experimental sample materials of SMW are primarily composed of water in the internal structure of the materials. This water can be ignored due to its low content compared with the liquid waste content of UMW; therefore, the actual moisture content values of medical waste in stricken areas should primarily refer to the analysis data of UMW. The inorganic materials of UMW are liquid medicine glass bottles and metallic instruments. The moisture (1.78%) of these materials is primarily derived from the residue on their inner walls. In UMW, the moisture of all types of component materials is mainly from all of the liquid ingredients during the use of the medical supplies. Thus, the moisture content of the medical waste from both stricken areas and base hospitals is primarily composed of all the types of liquid waste components introduced during the artificial use process. Because the UMW samples are produced by the similar cases generated from the base hospitals, the moisture content of the disaster medical waste generated from the relief unit should be close to that of UMW, which is 44.75%.

**Table 4:** Moisture content of SMW and UMW (%).

Category	Plastic	Biomass	Synthetic fibers	Rubber	Inorganic salts	Metals	Liquid waste	Moisture content of samples
SMW	0.15	3.74	0.75	0.46	5.68	0.03	—	1.50
UMW	4.84	58.52	16.62	24.86	1.78	1.85	100	44.75

In addition, it is worth mentioning that the moisture content varies greatly with the components of UMW. As shown by the data in Table 4, biomass is a highly sorptive medium with a moisture content of 58.52%, moderately sorptive media are synthetic fibers and rubber with moisture contents of 16.62% and 24.86%, respectively, and minimally sorptive media are plastic, inorganic salts and metals with moisture contents of 4.84%, 1.78% and 1.85%, respectively. In addition to liquid waste components, biomass is a main liquid waste carrier, primarily because most biomass medical supplies are cotton dressings, and their major ingredient is cellulose, which has excellent hygroscopicity and water absorption. In the process of diagnosis and treatment of injured persons, most body fluids from patients (including blood) and medical liquids and fluids are absorbed by and stored in gauze dressings, making them the largest moisture carrier in medical waste.

## 4. Conclusions

Garbage disposal started late in China compared with Japan, Germany and America, and there is limited experience in disaster medical waste disposal in this country. Given the actual live situation of earthquake disaster sites, it is difficult to sample and research medical waste at the source in these stricken areas. The study on composition, bulk density and moisture content of the medical waste generated from a medical relief unit is conducted by applying the RSR approach. The results from 35 cases showed that the medical waste generated from disaster relief consists of the following: plastic (43.2%), biomass (26.3%), synthetic fiber (15.3%), rubber (6.6%), liquid (6.6%), inorganic salts (0.3%) and metals (1.7%). The bulk density of medical relief waste is 249 kg/m^3^, and the moisture content is 44.75%. The data should be provided to assist storage, transportation, disposal and contamination control of medical waste in stricken areas. Through the study of the composition and characteristics of medical relief waste generated from disaster relief, we wish to introduce the RSR approach to readers and researchers. Furthermore, we hope more disaster relief agencies will become aware of the significance of medical case recording and storing because they may be important for the environmental evaluation of medical waste in disaster areas, as well as for medical waste management and disposal.

## Supporting Information

S1 Fig Report of a medical ethics review (in Chinese). (TIF)

S2 Fig Report of a medical ethics review (in English). (TIF)

S1 Table Bulk density data of SMW and UMW. (DOCX)

S2 Table Composition data of medical waste. (DOCX)
