# Supplementary figures and images for: Retrospection-Simulation-Revision: Approach to the Analysis of the Composition and Characteristics of Medical Waste at a Disaster Relief Site
Source: PLoS One. 2016 Jul 14;11(7):e0159261. doi: 10.1371/journal.pone.0159261 (PMC4944931; doi:10.1371/journal.pone.0159261)

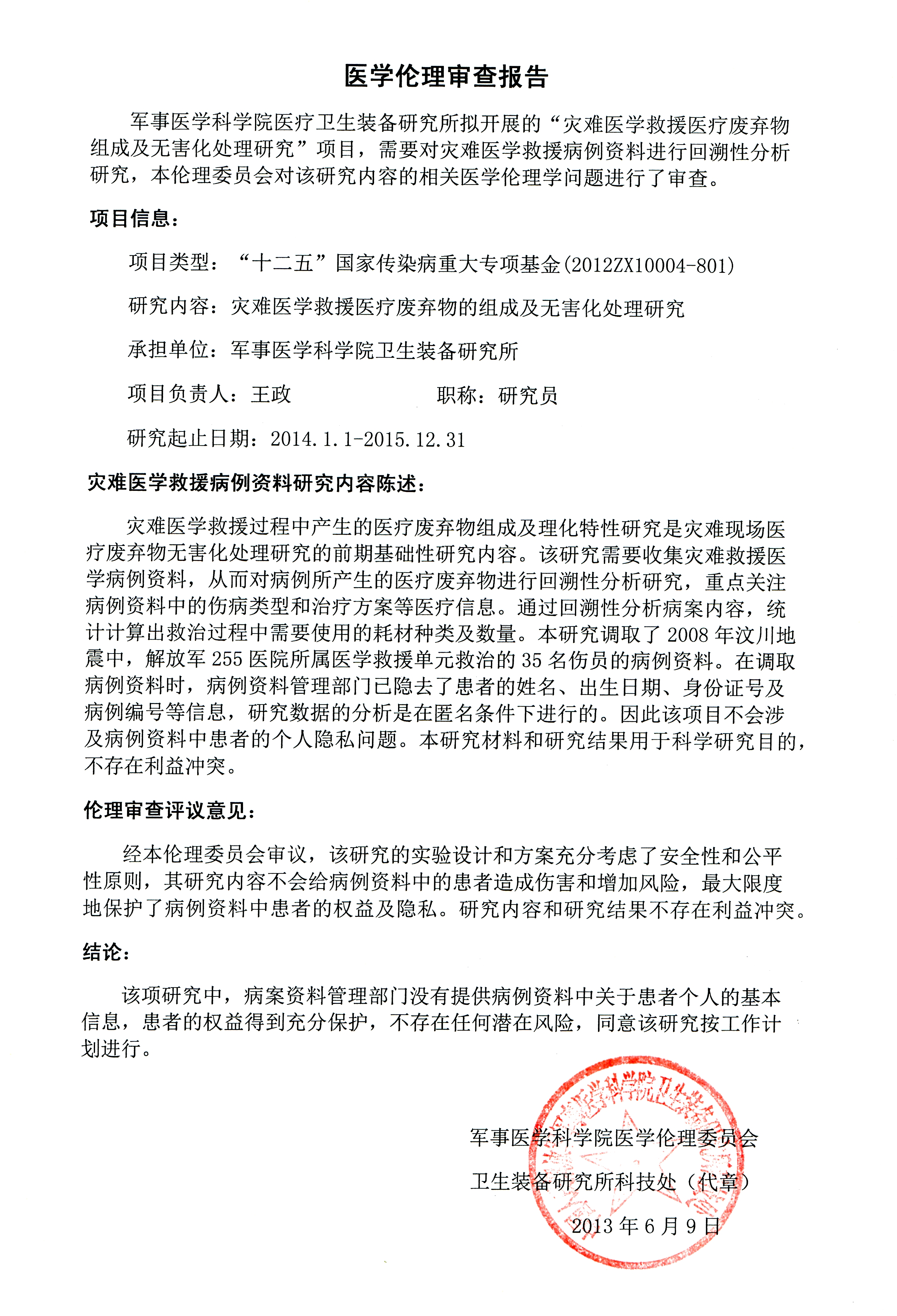

Supplement: S1 Fig — (TIF) [file pone.0159261.s001.tif]

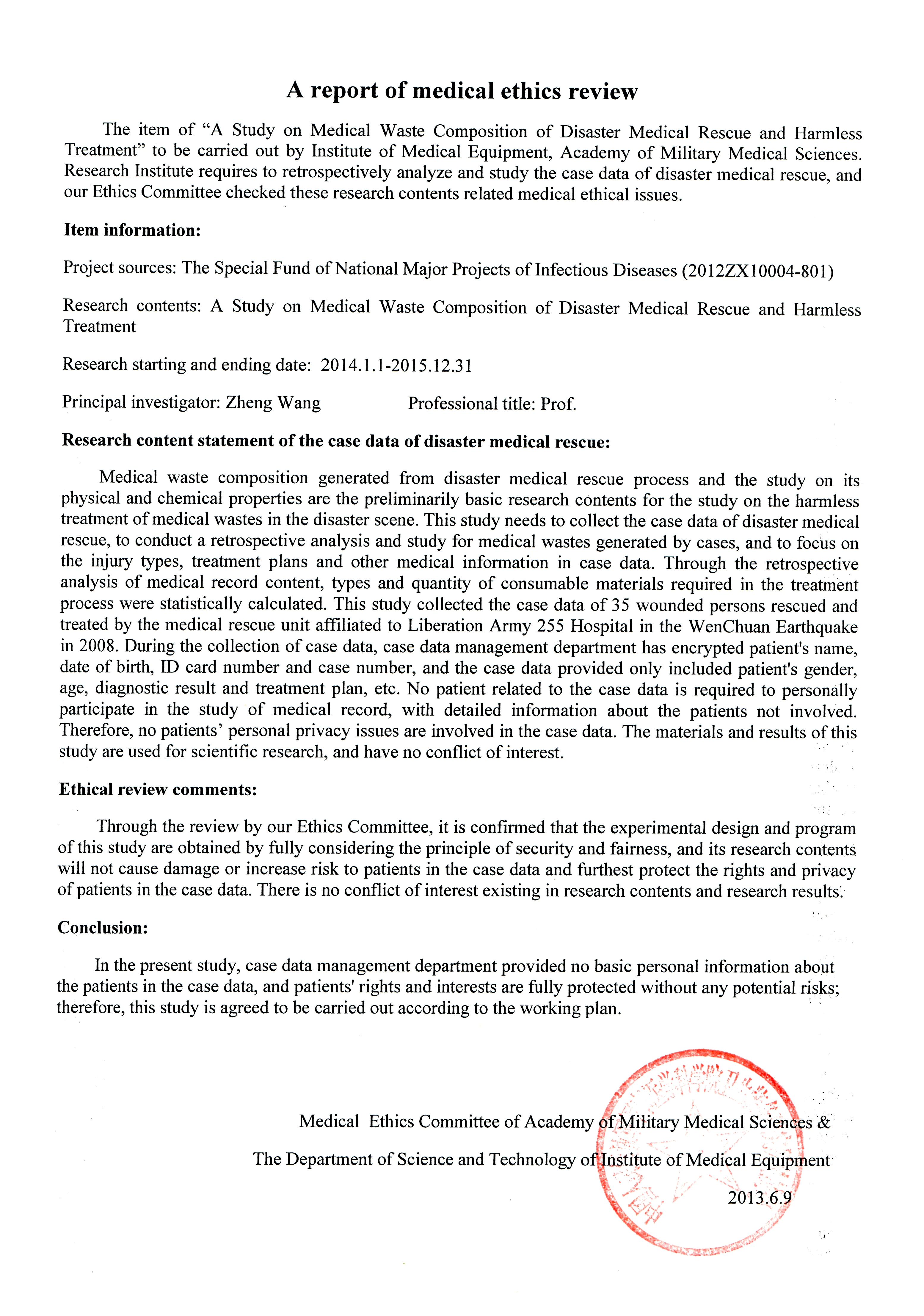

Supplement: S2 Fig — (TIF) [file pone.0159261.s002.tif]
