# Supplementary material for: Retrospection-Simulation-Revision: Approach to the Analysis of the Composition and Characteristics of Medical Waste at a Disaster Relief Site
Source: PLoS One. 2016 Jul 14;11(7):e0159261. doi: 10.1371/journal.pone.0159261 (PMC4944931; doi:10.1371/journal.pone.0159261)
Supplement: S1 Table — (DOCX) [file pone.0159261.s003.docx]

**S1 Table. The data of bulk densities of SMW and UMW**

| Sample | *V* (m^3^) | *M* (kg) | *M_j_* (kg) | | | | |  | *m* | *d* (kg/m^3^) |
| --- | --- | --- | --- | --- | --- | --- | --- | --- | --- | --- |
|  |  |  | *j=*1 | *j=*2 | *j=*3 | *j=*4 | *j=*5 |  |  |  |
| SMW | 0.050 | 1.70 | 10.88 | 11.06 | 10.39 | 10.48 | 10.98 |  | 5 | 181 |
| UMW | 0.050 | 1.70 | 14.02 | 14.21 | 14.77 | 14.18 | 13.66 |  | 5 | 249 |
