# Supplementary material for: Retrospection-Simulation-Revision: Approach to the Analysis of the Composition and Characteristics of Medical Waste at a Disaster Relief Site
Source: PLoS One. 2016 Jul 14;11(7):e0159261. doi: 10.1371/journal.pone.0159261 (PMC4944931; doi:10.1371/journal.pone.0159261)
Supplement: S2 Table — (DOCX) [file pone.0159261.s004.docx]

**S2 Table. Composition data of medical waste**

| Classification | | SMW | |  | UMW | |  | Revision |
| --- | --- | --- | --- | --- | --- | --- | --- | --- |
|  |  | *M*_i_ (kg)^*^ | *C*_i_ (%)^**^ |  | *M*_i_ (kg)^*^ | *C*_i_ (%)^**^ |  | *C_m_*_i_ (%)^**^ |
| Organic components | Plastic (i=1) | 5.85 | 46.3 |  | 3.18 | 16.3 |  | 43.2 |
|  | Biomass (i=2) | 3.55 | 28.1 |  | 11.29 | 57.7 |  | 26.3 |
|  | Synthetic fibers (i=3) | 2.08 | 16.4 |  | 2.14 | 10.9 |  | 15.3 |
|  | Rubber (i=4) | 0.89 | 7.1 |  | 1.35 | 6.9 |  | 6.6 |
| Inorganic components | Waste liquids (i=5) | ~~—~~ | ~~—~~ |  | 1.30 | 6.6 |  | 6.6 |
|  | Inorganic salts (i=6) | 0.03 | 0.3 |  | 0.15 | 0.8 |  | 0.3 |
|  | Metals (i=7) | 0.23 | 1.8 |  | 0.16 | 0.8 |  | 1.7 |
| Total (*M*) | | 12.63 | 100 |  | 19.57 | 100 |  | 100 |

* : wet basis

** : mass on a wet basis
